# Tempo and Mode of Diversification of Lake Tanganyika Cichlid Fishes

**DOI:** 10.1371/journal.pone.0001730

**Published:** 2008-03-05

**Authors:** Julia J. Day, James A. Cotton, Timothy G. Barraclough

**Affiliations:** 1 Department of Biology, University College London, London, United Kingdom; 2 Department of Zoology, The Natural History Museum, London, United Kingdom; 3 School of Biological and Chemical Sciences, Queen Mary, University of London, London, United Kingdom; 4 Division of Biology and NERC Centre for Population Biology, Imperial College London, Silwood Park Campus, Ascot, United Kingdom; University of Oxford, United Kingdom

## Abstract

**Background:**

Understanding the causes of disparities in species diversity across taxonomic groups and regions is a fundamental aim in evolutionary biology. Addressing these questions is difficult because of the need for densely sampled phylogenies and suitable empirical systems.

**Methodology/Principal Findings:**

Here we investigate the cichlid fish radiation of Lake Tanganyika and show that per lineage diversification rates have been more than six times slower than in the species flocks of Lakes Victoria and Malawi. The result holds even at peak periods of diversification in Lake Tanganyika, ruling out the age of the lake as an explanation for slow average rates, and is robust to uncertainties over the calibration of cichlid radiations in geological time. Moreover, Lake Tanganyika lineages, irrespective of different biological characteristics (e.g. sexually dichromatic versus sexually monochromatic clades), have diversified at similar rates, falling within typical estimates across a range of plant and animal clades. For example, the mostly sexually dichromatic haplochromines, which have speciated explosively in Lakes Victoria and Malawi, have displayed modest rates in Lake Tanganyika (where they are called Tropheini).

**Conclusion/Significance:**

Our results show that either the Lake Tanganyika environment is less conducive for cichlid speciation or the remarkable diversifying abilities of the haplochromines were inhibited by the prior occupancy of older radiations. Although the results indicate a dominant role for the environment in shaping cichlid diversification, differences in the timing of diversification among the Tanganyikan tribes indicate that biological differences were still important for the dynamics of species build-up in the lake. While we cannot resolve the timing of the radiation relative to the origin of the lake, because of the lack of robust geological date calibrations for cichlids, our results are consistent with a scenario that the different clades reflect independent adaptive radiations into different broad niches in the lake.

## Introduction

Explaining why some regions and taxonomic groups contain more species than others is a key goal of evolutionary biology. Different categories of explanations have been offered. For example, if resources place a limit on the number of species in a region, then diversity might often arise by adaptive radiation, in which lineages diversify rapidly to occupy different niches following the invasion of a new region or a shift in habitat usage [1], [2]. Alternatively, species richness may depend on rates of speciation and extinction: some organisms may evolve reproductive isolation more readily or have a lower risk of extinction than others, irrespective of any ecological limits on biomass [3]. Factors determining net diversification rates could include biological traits [4], [5], the environment [6]–[8], or the interaction between traits and the environment [7], [9].

One way to evaluate these alternatives is to explore the timing of diversification using molecular phylogenetics. For example, adaptive radiation predicts fast early diversification followed by slower rates of species-turnover once available niches have been filled [2]. Similarly, comparisons among taxa and regions can be used to test the effects of biological traits versus the environment [7]–[9]. Do diversification rates tend to be more similar between related taxa in different environments, between unrelated taxa in the same environments, or do they depend on an interaction between traits and environment? Such comparisons remain rare because of the need for phylogenetic trees containing nearly all species in the regions and taxa of interest.

Here, we use a near-complete phylogenetic tree of cichlid fish species from Lake Tanganyika (LT) to test these ideas in a lake fauna. Cichlids in the East African great lakes have provided classic examples of endemic radiations. Each lake harbors a species flock of many hundred species displaying astonishing levels of ecological, phenotypic and behavioural diversity. Phylogenetic and geological evidence indicates exceptionally rapid radiation from single ancestral species over very short evolutionary timescales in Lake Victoria (LV), 447–535 species and Lake Malawi (LM), 451–600 species [10], based on age estimates assuming near or total desiccation for these lakes at 12.4 Ka [11] and 1 Mya - 0.57 Ka [12], respectively. The same tribe, the haplochromines, has radiated in both lakes, where they comprise the majority of cichlid species. A major cause of the explosive radiations appears to be rapid speciation through sexual selection associated with shifts in male breeding coloration and associated female preferences e.g. [13]–[15].

The cichlid fauna of LT is also thought to have radiated rapidly [16], [17]. However, LT represents a very different scenario regarding its geological history and cichlid fauna. Despite being the oldest rift lake (9–12 Mya) [18], and larger than LM, it contains only one third as many cichlid species (200 reported in this study). LT has experienced fluctuations in lake level [19], but unlike LM and LV, is not thought to have experienced near or complete desiccation. The fauna derives from several distinct lineages (classified in up to 16 tribes [20]) derived from several invasions rather than from a single ancestor [21]. Many LT tribes are entirely sexually dichromatic, such as Cyprichromini, Bathybatini. Others are partially sexually dichromatic, such as Ectodini and Tropheini (the LT endemic haplochromines). Conversely, the most species rich lineage, Lamprologini (∼90–100 species) are sexually monochromatic. Therefore, LT provides the opportunity to compare the tempo of diversification among biologically distinct lineages occupying the same environment, and among members of the same lineage (the haplochromines) in different lakes.

In order to investigate patterns and rates of diversification within a clade, ideally nearly all the species should be sampled [22]. Previous comparisons have been limited by incomplete sampling and by a lack of statistical evaluation of expected patterns under hypothesized scenarios. To date, a single study [23], using lineage-through-time (LTT) plots, based on published linearised trees of five tribes (Limnochromini, Perissodini, Cyprichromini, Bathybatini, Ectodini) has attempted to evaluate patterns of LT cichlid diversification. The LTT plots of all five tribes were interpreted to show a speciation burst around the same time followed by a period of stasis, but the two most species rich tribes were not included and the conclusion was made from graphical interpretation rather than statistical analysis.

Compiling mtDNA sequence data for 152 (76%) endemic species, plus selected species from elsewhere [Table S1], we reconstruct the timing of diversification of the LT species flock using a Bayesian relaxed molecular clock approach. We also consider the potential effects of missing species on estimates of the timing and rate of diversification. We show that the tempo and mode of cichlid diversification is highly contingent on the environment. LT cichlids have diversified six times more slowly than endemic radiations in the other lakes, even during peak episodes of diversification. This result is robust to uncertainties in age of the cichlid flocks. Diversification rates are remarkably similar among LT tribes considering 95% confidence limits, despite variation in general niche (e.g. littoral versus deep-water) and breeding system (e.g. sexually monochromatic versus dichromatic). We do not find strong evidence for an initial rapid radiation followed by a uniform slow-down in rates. Instead, faster diversification occurred at intermediate periods, possibly coinciding with periods of changing lake levels e.g. [24], [25] or with successive invasions and separate adaptive radiations. The different tempo and mode of diversification in LT cichlids could reflect either its different physical environment or the constraining effects of earlier radiations on the remarkable diversifying abilities of the haplochromines.

## Results and Discussion

### Phylogeny and Age Estimates of the LT Cichlid Radiation

Maximum likelihood and Bayesian analyses recover trees with good support for relationships that are largely congruent with taxonomy (Figure S1, Text S1). The majority of species derive from a single well-supported clade (P, Figure S1), comprising two main lineages, the C-lineage [26], which contains several tribes including the Haplochromini, and a clade we call the L-lineage, which includes the Lamprologini and Eretmodini. The endemic Tanganyikan haplochromines, termed Tropheini, are closely related to the haplochromine radiations in LM and LV [21].

**Figure 1:**
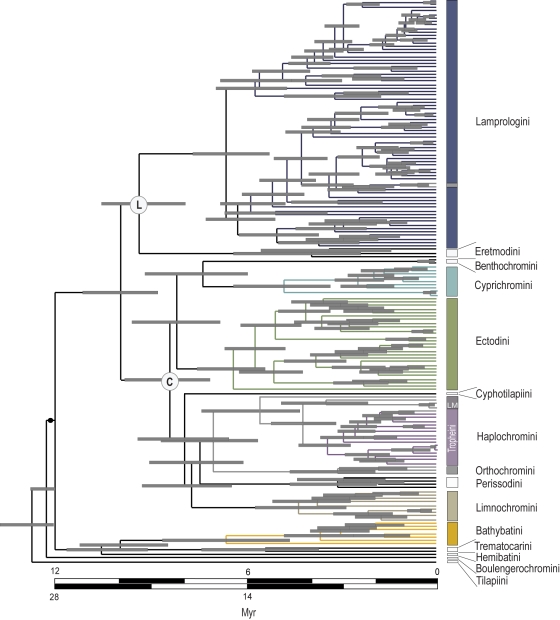
Phylogeny of LT cichlids reconstructed by Bayesian Inference and dated using a relaxed molecular clock (Yule speciation prior). Timescales for the two alternative calibrations are shown. The gray bars on each node indicate 95% credible intervals for the node ages. Black circle indicates the node used to date the tree. P = Principle lineage, C = C-lineage, L = L-lineage. Gray branches indicate non-endemic taxa. LM = Lake Malawi species.

Dating cichlid radiations in geological time is problematic because of the lack of fossils and other robust sources of independent calibration. Because of this uncertainty, we adopt, in turn, two widely different approaches used by previous authors. First, we use a date of 12 Mya for the root of the LT radiation (Figure 1), which is the proposed maximum geological age of LT [18] and which is similar to the estimated age for this node based on fossil cichlid calibrations [27]. Second, we use a date of 28 Mya for the same node based on calibrations of Gondwanan fragmentation for cichlids [27]. The cichlid timescale based on continental fragmentation supports a much older age (Cretaceous), of origin for cichlids, as opposed to the fossil record of the group, which dates back to the Eocene. This consequently has implications for the origin of LT cichlid faunas, in that Gondwanan dates imply the lake would have been colonised independently by the main lineages [27]–[29], rather than the accepted view based on the younger calibration, which assumes that early branching events occurred within the lake [27], [30]. We report results for both calibrations, but our main conclusions depend on relative dates, rather than absolute dates, and so are robust to these uncertainties. Dating the tree using a Bayesian relaxed molecular clock model in BEAST [31] resulted in the ultrametric consensus tree depicted in Figure 1.

### Net Diversification Rates in LT

**Figure 2:**
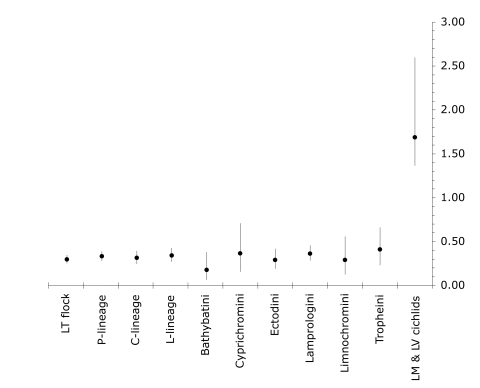
Average net diversification rates of LT cichlid clades (with missing species added) and the minimum rate estimate for LM and LV cichlid species flocks. Lines show the 95% confidence intervals incorporating both variation across sampled trees and the inherent stochasticity of the branching process.

We used models assuming constant per lineage rates of speciation and extinction to estimate average net diversification rates (speciation rate minus extinction rate) for the LT flock and constituent clades (Table S2) [32]. None of the clades displayed evidence for a non-zero extinction rate (Table S2). The average estimate of the per lineage diversification rate for the entire LT endemic flock, assuming a root age of 12 Mya and a Yule Prior for the distribution of node ages, is 0.31 species/Myr (we report per lineage rates throughout). The confidence interval incorporating both the stochastic nature of lineage branching and the uncertainty of relative date estimates across the Bayesian samples is 0.27 to 0.37 (Figure 2). To evaluate the effect of incomplete species sampling, we used taxonomic information about missing species to include them in our phylogeny. Each missing species was added to the tree in turn randomly with equal probability along the branches belonging to its likely clade (Figure S1 for placements). Addition of missing species increases the estimates to 0.36 (C.I. 0.30–0.40) for the entire flock (Table S2). We report results for the amended trees including the missing species but conclusions are qualitatively unaffected by their presence or absence. Our rate estimates are lower than previous ones for the LT flock, for example 0.9 [16] and 1.42 species/Myr [17]. Instead, they fall within the range of typical diversification rates estimated across a wide range of plant and animal taxa [5], [16], [33] [34]. Diversification rate estimates enforcing an alternative prior, proportional-to distinguishable (PD), are slower than those assuming the Yule prior e.g. 0.26 (C.I. 0.22–0.29) for the LT flock, but there remained no significant evidence for non-zero background extinction rates (Table S2). Similarly, assuming an older age of divergence of the LT radiation (28 Myr), rate estimates fall to 0.15 species/Myr (C.I. 0.13 to 0.17, Yule prior).

### Comparison with Lake Malawi and Lake Victora

Although the uncertainty in calibration dates prevents robust inference of absolute rates, we use the position of LM endemic species in our tree for a conservative comparison of diversification rates between LT and the LM and LV species flocks. We assume that the node separating the LM species from their nearest related LT lineage, i.e. their stem group age, defines an extreme maximum date, *t*, for the single common ancestor of the LM and LV species, which comprise a monophyletic group together with several riverine taxa [21]. Our approach, based on the younger calibration, uses an age of 3.1 Myr (C.I. 2.6 to 4.2) whereas the LV flock is believed to be much younger [e.g. 35]. We then estimate the net diversification rate as log(*N*)/*t*, where *N* is the number of species and the 95% confidence interval due to the stochasticity of the branching process is −log(1−0.025^1/*N*^)/*t* to −log(1−0.975^1/*N*^)/*t* [36]. Across the Bayesian samples, using the younger calibration yields an average diversification rate of 2.02 species/Myr (C.I. 1.16 to 3.12) with *N* = 898 (minimum species number for LM and LV flocks) [10], rising to 2.09 species/Myr (C.I. 1.71 to 3.19) with *N* = 1135 (maximum species number of LM and LV flocks) [10]. Using the older calibration yields average diversification rates of 0.87 (C.I. 0.70 to 1.33) and 0.90 (C.I. 0.73 to 1.37) respectively. The confidence intervals do not overlap with the C.I. of any of the LT clades (Figure 2). Therefore, the LM and LV species flocks have diversified at more than five times the rate of the LT flock. This conclusion applies even for the Tropheini, which are closely related to the LM and LV flocks and share their biological characteristics (see next section). Unlike previous comparisons, the conclusion is also independent of how we calibrated the radiations in geological time, because our comparisons derive from a single phylogenetic reconstruction, and is likely to be very conservative. Using a date for the LV flock of 0.12 Myr based on the older, conservative calibration [27] would yield a staggering per lineage rate estimate of over 50 species/Myr.

### Comparison of LT Cichlid faunas

Separate estimates of net diversification rate for the endemic tribes reveal a relatively uniform average speed of diversification among tribes when 95% confidence intervals are considered (Figure 2). Mean values for tribes ranged from 0.19–0.60 species/Myr, assuming 12 Myr calibration, falling to 0.07–0.24 species/Myr using the older calibration (Table S2). To test whether rates varied significantly among tribes, we multiplied the time period between successive nodes in each tribe by the number of lineages present during that interval [37]. Under a constant speciation rate model, these transformed internode distances are expected to be equal to the inverse of the diversification rate [36], [38]. An anova with clade as a factor revealed no significant difference in diversification rate either between the C and L lineages (*F_1,176_* = 0.1, *p*>0.5) or among tribes (*F_5,158_* = 1.6, *p*>0.1).

The fastest rates were recorded in the Tropheini (0.60 species/Myr) and Lamprologini (0.41 species/Myr), assuming a 12 Myr calibration, slowing to 0.16 and 0.24 species/Myr respectively, when the 28 Myr calibration is enforced. That these tribes share similar rates is of interest as they have contrasting evolutionary histories, with the former having a more recent origin than the latter, as well as displaying marked differences in breeding behavior. Lamprologines are substrate brooders and are monochromatic (∼5% of species exhibit extreme sexual dimorphism) [39]. Conversely, the Tropheini, as with all haplochromines, are mouthbrooders, and while most species are sexually dichromatic, some genera (e.g. *Tropheus*) are mainly monochromatic. The slowest rate is recorded in a deep-water tribe of strongly sexually dichromatic mouthbrooders, the Bathybatini, (irrespective of inclusion of the basal species, *B. minor*), with rates estimated from 0.19 – 0.08 species/Myr, assuming a 12 or 28 Myr calibration respectively. Therefore, contrary to previous findings (16), speciation is not uniformly faster in sexually dimorphic groups than in sexually monomorphic groups. The lamprologines also display much greater ecological diversity in trophic morphology and habitat than the Tropheini, consistent with a mode of ecological speciation [2]. Interestingly, a large proportion of lamprologines have exploited gastropod shells to live in and/or breed [40]. Occupation of this niche and the associated dwarfism it entailed may have triggered additional diversification (shell-brooding is significantly associated with increased diversification rate in the lamprologines (Table S1, Text S1). Whatever the mechanism of diversification in each tribe, it is clear that clades with very different biological characteristics have diversified to similar degrees in the lake.

### The Timing of Diversification

Adaptive radiation, driven by ecological opportunity, predicts rapid early speciation, followed by a subsequent slowdown [2], [41], whereas speciation by non-ecological sexual selection does not predict this trend [23], and may instead predict a speed up of net diversification rate, as recorded in LV cichlids [2], [after 42]. However, if several lineages colonized the lake and radiated independently, we might expect bursts of diversification following colonization but not necessarily congruent between different clades if they colonized at different times and filled different broad niches. Other scenarios might generate similar patterns, for example the appearance of new niches in the lake, such as shell beds, or if environmental events such as changing lake levels [24], [25] triggered an increased speciation rate [43].

**Figure 3:**
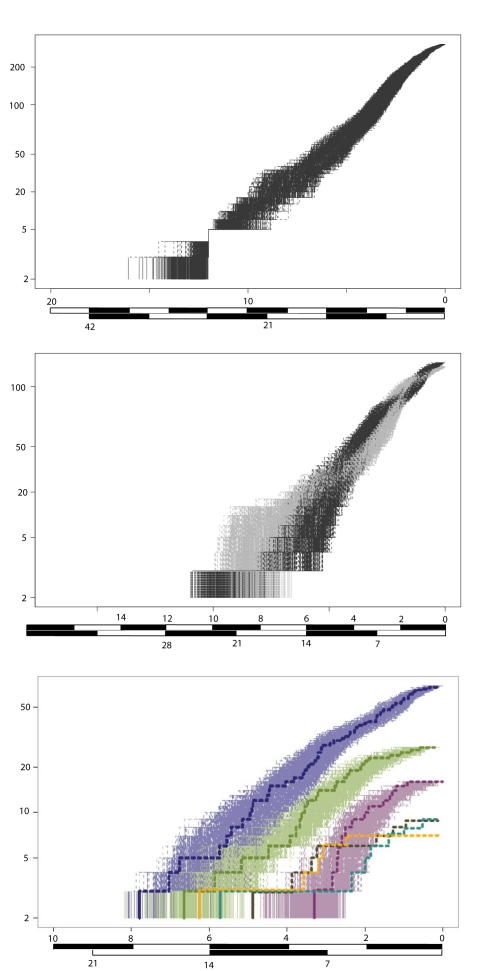
Lineage-through-time (LTT) plots, including missing species, based on 1000 sampled Bayesian trees: a) LT radiation (black); b) L-lineage (black) and C-lineage (gray); c) tribes: Lamprologini (purple), Ectodini (green), Tropheini (plum), Limnochromini (fawn), Cyprichromini (aqua), Bathybatini (yellow). A single LLT plot is highlighted for all tribes, but is only displayed for the latter three tribes for clarity. Timescales for the two alternative calibrations are shown.

While distinguishing all these scenarios is difficult, we test whether diversification rates have declined over time and whether any changes have been synchronous among clades. Lineage-through-time (LTT) plots show that rates have slowed down somewhat: a straight line is expected if there has been a constant speciation rate with no extinction (Figure 3) [32]. Under both priors (Yule and PD), a significant decline is recorded (Pybus and Harvey **γ** statistic [44]) for the entire endemic flock (Figures 3a, S6a, **γ** = −2.25, *p* = 0.06; S6e -4.25, *p* = 0.000) and the principle radiation (Figures 3a, S6a, **γ** = −3.20, *p* = 0.007; S6e -4.52, *p* = 0.000). This is also true for the L-lineage under the PD prior (S6e -3.55, *p* = 0.002), while the decline enforcing the Yule prior is marginally non-significant (Figure 3b, S6a, **γ** = −2.77, *p* = 0.02). Among the tribes only the Lamprologini (which comprise the majority of the L-lineage) display a significant decline in rates over time (Figure 3c, Table S2). Declines among the other tribes and C-lineage are non-significant (Table S2) and overall there is not the strong plateau expected of an early adaptive radiation and slow-down model. Instead, the curve for the C-lineage is more sigmoid in shape (Figure 3b).

**Figure 4:**
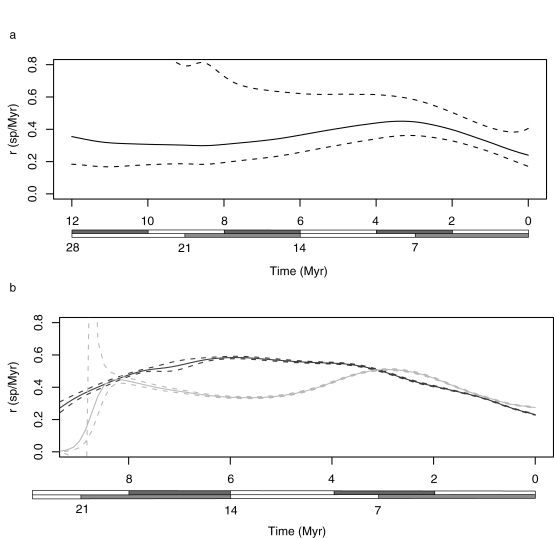
Net diversification rate through time fitted by General Additive Models (GAMs) for a) the entire flock and b) the L- (black) and C- (gray) lineages. Scale bars (x-axis) represent both 28 Myr and 12 Myr calibrations and the y-axis is the per lineage diversification rate in species per million years. Dashed lines show standard errors combining those across Bayesian samples and those due to uncertainty in the model in a), but only those across Bayesian samples in b). In the latter case, standard errors due to uncertainty in the model are extremely large.

General Additive Models (GAM) were used to explore the net change in diversification rates over time in more detail (Figure 4) [45]. These show that, across the entire flock, diversification rates were highest at intermediate periods, around 3 to 4 Mya (12 Myr calibration) or 7 to 8 Mya (28 Myr calibration, Figure 4a). These findings appear to contradict those of the Pybus and Harvey test [44], which implied a significant slowdown. While the robustness of GAMs has been questioned in the past, in this case similar conclusions are reached from trees derived from the two different priors of node age distributions and from the repeated samples from each MCMC analysis. Therefore, we believe the GAMs are fitting real features of the distribution of node ages in our sample: the average overall decline in diversification rate is largely due to decline from intermediate to recent periods, which encompass most of the nodes contributing information to the test statistics.

The pattern differs marginally between the C- and L-lineages (ANOVA comparing GAMs with clade as a factor or not, median F across Bayesian samples = 2.1, median *p* = 0.033). The C-lineage is typified by an early and late peak in diversification rates, separated by a period of low net rates, during which time the L-lineage sustained higher net rates (Figure 4b). Both lineages reveal a final decline in rates towards the present, which could reflect either a true decline, over-conservative placement of the species missing from our sample, or a taxonomic artifact of failure to recognize recently diverged species. If clades have different peak periods of diversification, even though average diversification rates are relatively similar among tribes, this would suggest that biological differences among tribes did matter in the build-up of present-day diversity. Either clades were affected differentially by different environmental events or they filled broadly different niches in the lake. If biological differences were irrelevant, we would have expected any changes in diversification rate to be congruent among the different clades. However, even with a near complete sample of species, and potentially large differences in average diversification rate at given times, the differences between the two clades are only marginally significant in our present analyses.

Interpreting the causes of the diversification pulses is even more difficult. With the younger calibration based on lake age and fossils, then the first peak in the C-lineage corresponds roughly with the onset of full lacustrine conditions and the second peak with a shift in global climate and the aridification of Africa [24], when lake levels are assumed to have dropped. With the older calibration based on Gondwanan fragmentation, then the initial peak would correspond to an early radiation generating the ancestors of the different tribes, perhaps in a now extinct lake [23], and the second peak with the onset of full lacustrine conditions. Similarly, the L-lineage may have sustained high rates from basin formation to the onset of full lacustrine conditions (older calibration) or after these events (younger calibrations). The older calibration is appealing because peak diversification across the entire flock (ca. 8 to 9 Mya) would then be congruent with basin formation and development, but this requires that diversification leading to the different tribes occurred at an earlier time. However, the older calibration leads to rather slow estimates of net diversification rates among LT cichlids (although not LM and LV). Clearly, further evidence is needed to resolve these alternatives. Given the difficulty in finding robust calibrations for cichlids, and the limits of sample size being reached for distinguishing alternative diversity models, one possibility would be to compare the timing of diversification across a broad suite of LT endemic taxa, especially those with fossil records or alternative geological calibrations e.g. [46], [47].

We conclude that LT cichlid fishes have diversified much more slowly than those of LV and LM, even the endemic LT haplochromines. In contrast to LM and LV, the LT species flock derives from a prolonged accumulation of species, rather than rapid, recent radiation. Clearly, the environment plays a major role in determining cichlid diversification rates. One possibility is that the physical environment of LT is less conducive for cichlid speciation, perhaps by inhibiting whatever special mechanism causes fast speciation in LM and LV. Turbid waters have been shown to impede sexual selection in LV [48], raising the possibly that a similar phenomenon may have occurred during the history of LT, although currently these waters are very transparent [49]. Alternatively, the remarkable diversifying abilities of haplochromines could have been inhibited because the lake was already occupied by older radiations, such as the lamprologines and ectodines, which occupy similar habitats. The similarity of net diversification rates among tribes with very different biological characteristics might seem to imply that traits had minimal influence on the progress of diversification in the lake. However, there is some evidence that clades did vary in the timing of peak rates of diversification: lineage identity was important for determining diversification potential over large periods of the lake's history. Whether these differences reflect differential responses to episodes of climate change or successive adaptive radiations into different broad niches is difficult to resolve until more robust calibrations are available. However, a plausible explanation is that the lake was colonized several times leading to a series of independent adaptive radiations into different broad niches followed by a slow-down in rate in each descendent clade.

## Materials and Methods

### Phylogeny

Previous phylogenetic studies of LT cichlids consider subsets of the data (< one third of sampled species) [26], [30]. However, recent proliferation of sequence data represents an important source for comparative studies on LT cichlids. Densely sampled trees are important in diversification studies as well as providing information on relative timing of cladogensis [22]. Increased taxon sampling has been shown to improve both the precision and accuracy of phylogenetic reconstruction [50]–[51]. We reconstruct the evolutionary relationships of 161 cichlid species (152 LT endemics, 9 non-endemics), representing 76% of all LT species (assuming a LT cichlid flock of 200 endemic species, Table S1), based on two mtDNA genes: the protein coding, ND2 (∼1047 bp) and the non-coding, control region (CR), (partial sequence data of 347 bp from the 5′-end) using published sequence data (Table S1). Alignment of ND2 sequences was unambiguous, in comparison to the CR, which varied slightly in length among sequences (337-335, with the longest gap consisting of a three bp indel). The CR data was initially aligned using CLUSTAL X [52] and subsequently both datasets were optimised by visual inspection in a manual alignment editor [53]. Modeltest v.3.7 [54] selected the evolutionary model GTR+I+Γ, using Akaike Information Criteria and was implemented in subsequent analyses. Bayesian Inference, using Metropolis-coupled Markov Chain Monte Carlo (MCMCMC), implemented in MrBayes v.3.1.2 [55], was run for 2×10^6^ generations, sampling every 100 generations (four chains, temperature 0.2), to ensure convergence, with the first 250,000 generations discarded as burn-in. This analysis was partitioned to account for the different behaviour of the genes as well as the third codon of ND2. Maximum likelihood inference was implemented in GARLI v.0.942 (Genetic Algorithm for Rapid Likelihood Inference) [56] also sampling 2×10^6^ generations for multiple runs to ensure similar trees and lnL scores. Branch support was evaluated using non-parametric bootstrapping (BS) consisting of 1000 pseudoreplicates (using GARLI), and Bayesian posterior probabilities (BPP). The tree topologies recovering using Bayesian Inference and Maximum Likelihood were evaluated using the approximately unbiased test [57], implemented in the program CONSEL [58] using the site likelihood scores from PAUP* [59] see Text S1.

### Molecular Dating

A likelihood ratio test [60] performed on the concatenated data set [molecular clock enforced (-ln L 41653.91), not enforced (-ln L 41352.23), *χ* ^2^ = 603.36, df = 157, *p* = 0.00,)] rejected overall constancy of rates of evolution. Divergence time analyses using a log-normal distributed relaxed molecular clock [31] were performed using BEAST v1.4.6 [61], which uses Bayesian inference and an MCMC procedure to estimate the posterior distribution of rates and times. We used a constant-rate Yule (speciation process) prior, and all other priors and operators were the default settings, except that the root was constrained with a point prior, and the starting tree was the maximum posterior probability topology form the Bayesian phylogenetic analysis, and this topology remained fixed throughout the MCMC. Three independent chains were run for 2×10^6^ generations each, and convergence checked visually by comparing these runs after a burn-in of 2×10^5^ generations was discarded. As there is no Bayesian MCMC implementation of speciation models that accommodate extinction, we also performed the same analyses using a Proportional-to-distinguishable (PD) prior. The PD arrangements prior puts an equal probability on every different labelled tree [62]. To investigate the sensitivity of divergence time estimates, we performed the same analyses using exponential relaxed clock and strict clock models in BEAST. Results from these analyses were largely congruent with those from the analysis reported here.

### Estimating Diversification Rates

The resulting ultrametric trees obtained using both Yule and PD priors using a log-normal distributed relaxed clock calculated by BEAST were imported into the APE 1.2–2 package [63] of the R statistical programming language [64] to generate semi logarithmic LLT plots calculated for 1000 sampled trees (sampling every 2500 generations of the LT radiation and across different taxonomic grouping of LT cichlids We also consider diversification rate (speciation minus extinction rates) using the results from BEAST, implementing different constant speciation and extinction rate models using the birthdeath function in APE, for the same taxonomic groups, similarly calculated from 1000 sampled trees (sampling every 2500 generations). All non-endemic taxa were excluded from these analyses. For each clade we also tested for significant departures from the constant speciation model using the γ statistic [44]. Positive values for this statistic signify that there has been an apparent increase in diversification rate towards the present, whereas negative values indicate a deceleration in diversification rate towards the present. Generalized additive models were constructed using the mgcv library in R [64]. Models of transformed internode intervals as a function of time were fitted assuming a basis dimension (k) of 50 knots. Unbiased risk estimation (UBRE) was then used to estimate smoothing parameters and the effective degrees of freedom for the smooth term [65]. Hypothesis-testing comparing a single and different smoothing functions for the C and L lineages was based on unpenalized GAMs [65]; for these, *k* was set to 10 because statistical power falls as *k* is increased. Nested models (with a single smoothing function or a separate smoothing functions for the two lineages) were compared using ANOVA and F tests. We further applied these models to the data from the PD prior, but found the result was non-significant.

### Web Resources

URLs for data presented herein are as follows: GenBank, http://www.ncbi.nih.gov/Genbank/index.html

**Table 1:** Lake Tanganyika cichlid clades, including life history traits and age estimates (mean and 95% confidence intervals), based on 12 and 28 Myr calibrations [27] using a Bayesian relaxed-clock model enforcing the Yule prior [31].

Taxonomic grouping	*N* species	Mode of life	Habitat	Parental care	Calibration (Myr)	
					12	28
LT radiation	152/200	B/P	L/D	MB/SB		
P-radiation	137/178	B/P	L/D	MB/SB	10.0 (8.7–11.1)	23.3 (20.3–25.9)
Trematocarini	2/9	B	D	MB	9.9 (8.3–11.2)	23.1 (19.3–26.1)
L-lineage	72/93	B	L	MB/SB	9.3 (7.9–10.8)	21.7 (18.4–25.2)
C-lineage	65/85	B/P	L/D	MB	8.4 (8.0–10.6)	19.6 (18.7–24.7)
Bathybatini	7/7	P	D	MB	6.6 (4.6–9.2)	15.4 (10.7–21.5)
Lamprologini	69/87	B	L (s, r, sh)	SB	6.6 (5.4–7.9)	15.4 (12.6–18.4)
Ectodini	27/31	B	L (s, r)	MB	6.4 (5.4–7.5)	14.9 (12.6–17.5)
Cyprichromini	9/9	P	L	MB	4.8 (3.3–6.5)	11.2 (7.7–15.2)
Limnochromini	9/10	B	D	MB	4.3 (3.0–6.1)	10.0 (7.0–14.2)
Eretmodini	3/6	B	L (r)	MB	3.9 (2.1–6.4)	9.1 (4.9–14.9)
Tropheini	16/26	B	L (r)	MB	3.4 (2.7–4.5)	7.9 (6.3–10.5)
Perissodini	4/9	P	D	*	3.1 (1.8–4.6)	7.2 (4.2–10.7)

*N* = number of endemic species included in this study/number of reported endemic species (Table S2). P = pelagic, B = benthic, L = littoral, D = deepwater (habitats: r = rocky, s = sandy, sh = shells), MB = mouth-brooding, SB = substrate brooding, * transitional between SB and MB

## Supporting Information

Figure S1 Phylogeny of Lake Tanganyika cichlids based on mtDNA. Bayesian tree recovered from combined analysis of ND2 and control region datasets. Branch support indicated by BPP and BS derived from BI and ML methods (>50%) from left to right respectively. * indicates the same score for both support methods. P = principal lineage, C = C-lineage, L = L-lineage, LM = Lake Malawi. Gray branches indicate non-endemic taxa. Gray circles containing numerical numbers, indicate number of additional species added at that branch, closed gray circle indicates no additional species added beyond that node. Black circle indicates the node used to calibrate the tree. Full details for all species used in this study, including GenBank accession numbers are provided in Table S2.
(0.61 MB EPS)

Table S1 Cichlid taxa included in phylogenetic analyses and associated Genbank numbers.
(0.13 MB PDF)

Table S2 Speciation rate statistics for the LT cichlid radiation, major lineages and constituent tribes.
(0.08 MB PDF)

Text S1 Lake Tanganyika cichlid phylogeny Key-innovation test Supporting references
(0.07 MB PDF)
