# Supplementary material for: Tempo and Mode of Diversification of Lake Tanganyika Cichlid Fishes
Source: PLoS One. 2008 Mar 5;3(3):e1730. doi: 10.1371/journal.pone.0001730 (PMC2248707; doi:10.1371/journal.pone.0001730)
Supplement: Table S1 — Cichlid taxa included in phylogenetic analyses and associated Genbank numbers. (0.13 MB PDF) [file pone.0001730.s002.pdf]

Table S1. Cichlid taxa included in phylogenetic analyses and associated Genbank numbers. Sequences were obtained from published sources [1]–[13], [Supporting Text] *N* = number of species included in this study / number of reported endemic species. In addition to the 152 species used to construct the phylogeny, a further 48 possible species (highlighted in gray) are described or mentioned in the literature. † denotes three additional ‘species’ of Eretodini that are included as missing species, after previous studies [14] that reveal six mitochondrial lineages for this group. The total of 200 endemic cichlid species is higher than previously estimated (162-184) [15]. Missing species, are subsequently inserted into the phylogeny based on their taxonomy (see Figure S1 for placements). Lamprologine species that have divergent placements within the tree (Figure S1) are distinguished by the suffix I and II. This might be the result of taxonomic misidentification or introgression [3]. For the purposes of this study, they are considered as separate taxonomic units; △ denotes shell-brooding lamprologine taxa; LM, Lake Malawi; LT, Lake Tanganyika; LV, Lake Victoria.

Taxonomy				GenBank accession numbers	
**Tribe**	***N***	**Species**	**Locality**	**ND2**	**Control Region**
**Bathybatini**	7/7	*Bathybates fasciatus*	LT	AY663734 [7]	AY663780 [7]
		Bathybates ferox	LT	AY663737 [7]	AY663783 [7]
		Bathybates graueri	LT	AY663726 [7]	AY663771 [7]
		Bathybates horni	LT	AY663735 [7]	AY663781 [7]
		Bathybates leo	LT	AY663731 [7]	AY663776 [7]
		Bathybates minor	LT	AY663722 [7]	AY663767 [7]
		Bathybates vittatus	LT	AY663728 [7]	AY663773 [7]
**Benthochromini**	2/2	*Benthochromis melanoides*	LT	AY682513 [4]	AY682476 [4]
		Benthochromis tricoti	LT	AY682515 [4]	AY682478 [4]
**Boulengerochromini**	1/1	*Boulengerochromis microlepis*	LT	AF317229 [5]	AY929939 [10]
**Cyphotilapiini**	1/1	*Cyphotilapia frontosa*	LT	CFU07247 [8]	AF400732 [9]
**Cyprichromini**	9/9	*Cyprichromis leptosoma*	LT	AY337786 [5]	AY339053 [5]
		Cyprichromis cf *leptosoma I*	LT	AY740379 [1]	AY740328 [1]
		Cyprichromis cf *leptosoma II*	LT	AY740355 [4]	AY740304 [4]
		Cyprichromis cf *leptosoma III*	LT	AY740344 [4]	AY740290 [4]
		Cyprichromis microlepidotus	LT	AY740354 [4]	AY740301 [4]
		Cyprichromis pavo	LT	AY740358 [1]	AY740307 [1]
		Cyprichromis zonatus	LT	AY740347 [1]	AY740295 [1]
		Paracyprichromis brieni	LT	AY740352 [1]	AY740299 [1]
		Paracyprichromis nigripinnis	LT	AY740339 [1]	AY740283 [1]
**Ectodini**	27/31	*Asprotilapia leptura*	LT	AY337772 [6]	AY400701 [9]
		Aulonocranus dewindti	LT	AY337782 [6]	AY339007 [6]
		Callochromis macrops	LT	AY337795 [6]	AY339051 [6]
		Callochromis melanostigma	LT	n.a.	n.a.
		Callochromis pleurospilus	LT	AY337771 [6]	Z21735 [13]
		Callochromis stappersii	LT	AY337775 [6]	AY339048 [6]
		Cardiopharynx schoutedeni	LT	AY337791 [6]	AY339000 [6]
		Cunningtonia longiventralis	LT	AY337780 [6]	AY338999 [6]
		Cyathopharynx furcifer	LT	AY337781 [6]	AY338981 [6]
		Ectodus descampsii	LT	AY337790 [6]	AY339014 [6]
		Ectodus cf. descampsii	LT	n.a.	n.a.
		Enantiopus melanogenys	LT	AY337770 [6]	AY339022 [6]
		Grammatotria lemairii	LT	AY337787 [6]	AY339018 [6]
		Lestradea perspicax	LT	AY337765 [6]	Z21745 [13]
		Lestradea stappersii	LT	AY337792 [6]	AY338997 [6]
		Microdontochromis rotundiventralis	LT	AY337793 [6]	AY339021 [6]
		Microdontochromis tenuidentatus	LT	AY337784 [6]	AY339019 [6]
		Ophthalmotilapia boops	LT	AY337773 [6]	AY338987 [6]
		Ophthalmotilapia heterodonta	LT	n.a.	n.a.
		Ophthalmotilapia nasuta	LT	AY337783 [6]	AY338989 [6]
		Ophthalmotilapia ventralis	LT	AY337774 [6]	AY338993 [6]
		Xenotilapia bathyphila	LT	AY337789 [6]	AY339028 [6]
		Xenotilapia cf *bathyphila*	LT	AY337768 [6]	AY339026 [6]
		Xenotilapia boulengeri	LT	n.a.	n.a.
		Xenotilapia caudafasciata	LT	AY337777 [6]	AY339035 [6]
		Xenotilapia flavipinnis	LT	AY337794 [6]	AY339034 [6]
		Xenotilapia longispinis	LT	AY337779 [6]	AY339037 [6]
		Xenotilapia ochrogenys	LT	AY337767 [6]	Z21750 [13]
		Xenotilapia sima	LT	AY337785 [6]	AY339039 [6]
		Xenotilapia spiloptera	LT	AY337788 [6]	AY339043 [6]
		Xenotilapia papilio (‘sunflower’)	LT	AY337776 [6]	AY339045 [6]
**Eretmodini**	3/6†	*Eretmodus cyanostictus*	LT	DQ055010 [11]	DQ054911 [11]
		Spathodus erythrodon	LT	DQ055008 [11]	DQ054909 [11]
		Tanganicodus irsacae	LT	DQ055007 [11]	DQ054908 [11]
**Haplochromini**		*Astatoreochromis alluaudi*	LV region	AY930071 [10]	AY929966 [10]
		Astatotilapia burtoni	LT	AY930060 [10]	AY929955 [10]
		Melanochromis auratus	LM	AY930069 [10]	AY929964 [10]
		Metriaclima zebra	LM	DQ093114 [2]	AY930025 [10]
**Hemibatini**	1/1	*Hemibates stenosoma*	LT	AY663719 [7]	AY663764 [7]
**Lamprologini**	69/87	*Altolamprologus calvus*	LT	EF462256 [3]	EF462318 [3]
		Altolamprologus compressiceps △	LT	EF462257 [3]	EF462319 [3]
		Altolamprologus fasciatus	LT	EF462255 [3]	EF462317 [3]
		Chalinochromis brichardi	LT	EF462232 [3]	EF462294 [3]
		Chalinochromis popeleni	LT	U07244 [8]	n.a.
		Julidiochromis dickfeldi	LT	EF462230 [3]	EF462292 [3]

Julidiochromis marlieri	LT	EF462227 [3]	EF462289 [3]
Julidiochromis ornatus	LT	EF462229 [3]	EF462291 [3]
Julidiochromis regain	LT	EF462228 [3]	EF462290 [3]
Julidiochromis transcriptus	LT	EF462231 [3]	EF462293 [3]
Lamprologus callipterus Δ	LT	EF462258 [3]	EF462320 [3]
Lamprologus congoensis	Congo river	AY740385 [4]	AF400719 [9]
Lamprologus finalimus	LT	n.a.	n.a.
Lamprologus kungweensis Δ	LT	n.a.	n.a
Lamprologus laparogramma Δ	LT	EF462278 [3]	EF462340 [3]
Lamprologus lemairii	LT	EF462271 [3]	EF462333 [3]
Lamprologus ocellatus Δ	LT	EF462259 [3]	EF462321 [3]
Lamprologus ornatipinnis Δ	LT	EF462260 [3]	EF462322 [3]
Lamprologus signatus Δ	LT	n.a.	n.a.
Lamprologus speciosus Δ	LT	DQ055032 [11]	DQ054934 [11]
Lamprologus stappersi Δ	LT	n.a.	n.a.
Lamprologus teugelsi	Congo river	AF398225 [9]	AF400720 [9]
Lepidolamprologus attenuatus I Δ	LT	EF462274 [3]	EF462336 [3]
Lepidolamprologus attenuatus II Δ	LT	DQ055036 [11]	DQ054912 [11]
Lepidolamprologus boulengeri Δ	LT	EF462254 [3]	EF462316 [3]
Lepidolamprologus cunningtoni	LT	DQ055017 [11]	DQ055017 [11]
Lepidolamprologus elongates	LT	EF462268 [3]	EF462330 [3]
Lepidolamprologus hecqui Δ	LT	DQ055018 [11]	DQ054920 [11]
Lepidolamprologus kendalli	LT	EF462269 [3]	EF462331 [3]
Lepidolamprologus meeli I Δ	LT	EF462277 [3]	EF462339 [3]
Lepidolamprologus meeli II Δ	LT	DQ055051 [11]	DQ054950 [11]
Lepidolamprologus meleagris Δ	LT	DQ055027 [11]	DQ054929 [11]
Lepidolamprologus nkambee	LT	EF462270 [3]	EF462332 [3]
Lepidolamprologus pleuromaculatus	LT	n.a.	n.a.
Lepidolamprologus profundicola	LT	DQ055025 [11]	DQ054927 [11]
Lepidolamprologus cf *profundicola*	LT	EF462275 [3]	EF462337 [3]
Lepidolamprologus variostigma	LT	EF462253 [3]	EF462315 [3]
Neolamprologus bifasciatus	LT	EF462240 [3]	EF462302 [3]
Neolamprologus brevis Δ	LT	EF462263 [3]	EF462325 [3]
Neolamprologus brichardi I	LT	EF462245 [3]	EF462307 [3]
Neolamprologus brichardi II	LT	DQ055015 [11]	DQ054917 [11]
Neolamprologus buescheri	LT	EF462243 [3]	EF462305 [3]
Neolamprologus calliurus Δ	LT	n.a.	n.a.
Neolamprologus caudopunctatus Δ	LT	EF462272 [3]	EF462334 [3]
Neolamprologus christyi I	LT	EF462226 [3]	EF462288 [3]
Neolamprologus christyi II	LT	DQ055058 [11]	DQ054954 [11]
Neolamprologus crassus	LT	n.a.	n.a.
Neolamprologus cylindricus	LT	EF462224 [3]	EF462286 [3]
Neolamprologus falicula	LT	EF462246 [3]	EF462308 [3]
Neolamprologus fasciatus	LT	n.a.	n.a.
Neolamprologus furcifer	LT	EF462249 [3]	EF462311 [3]
Neolamprologus gracilis	LT	n.a.	n.a.
Neolamprologus helianthus	LT	DQ055013 [11]	DQ054915 [11]
Neolamprologus leleupi	LT	EF462251 [3]	EF462313 [3]
Neolamprologus leloupi	LT	EF462250 [3]	EF462314 [3]
Neolamprologus longicaudatus	LT	EF462250 [3]	EF462312 [3]
Neolamprologus longior	LT	n.a.	n.a.
Neolamprologus marunguensis	LT	DQ055014 [11]	DQ054916 [11]
Neolamprologus modestus	LT	DQ055012 [11]	DQ054914 [11]
Neolamprologus mondabu Δ	LT	EF462241 [3]	EF462303 [3]
Neolamprologus moori	LT	EF462218 [3]	EF462280 [3]
Neolamprologus multifasicatus Δ	LT	EF462265 [3]	EF462327 [3]
Neolamprologus mustax	LT	EF462223 [3]	EF462285 [3]
Neolamprologus niger	LT	AY740391 [4]	n.a.
Neolamprologus obscurus	LT	n.a.	n.a.
Neolamprologus olivaceous	LT	AY740393 [4]	AY438783 [9]
Neolamprologus pectoralis	LT	EF462238 [3]	EF462300 [3]
Neolamprologus prochilus	LT	EF462248 [3]	EF462310 [3]
Neolamprologus pulcher I	LT	EF462244 [3]	EF462306 [3]
Neolamprologus pulcher II	LT	AY740395 [4]	n.a.
Neolamprologus savoryi	LT	EF462247 [3]	EF462309 [3]
Neolamprologus schreyeni	LT	n.a.	n.a.
Neolamprologus sexfasciatus	LT	n.a.	n.a.
Neolamprologus similis Δ	LT	DQ055030 [11]	DQ054932 [11]
Neolamprologus splendens	LT	n.a.	n.a.
Neolamprologus tetracanthus	LT	EF462220 [3]	EF462282 [3]
Neolamprologus toae	LT	EF462222 [3]	EF462284 [3]
Neolamprologus tretrocephalus	LT	EF462219 [3]	EF462281 [3]
Neolamprologus wauthioni	LT	n.a.	n.a.
Neolamprologus ventralis	LT	EF462233 [3]	EF462295 [3]
Telmatochromis bifrenatus Δ	LT	EF462235 [3]	EF462297 [3]
Telmatochromis brachygnathus Δ	LT	n.a.	n.a.
Telmatochromis brichardi Δ	LT	EF462236 [3]	EF462298 [3]
Telmatochromis burgeoni Δ	LT	n.a.	n.a.
Telmatochromis dhonti Δ	LT	n.a.	n.a.
Telmatochromis temporalis Δ	LT	EF462234 [3]	EF462296 [3]

		Telmatochromis vittatus	LT	EF462237 [3]	EF462299 [3]
**Limnochromini**	9/10	*Greenwoodochromis bellcrossi*	LT	AY682523 [4]	AY682486 [4]
		Greenwoodochromis christyi	LT	AY682525 [4]	AY682488 [4]
		Baileychromis centropomoides	LT	AY682509 [4]	AY682472 [4]
		Gnathochromis permaxillaris	LT	AY682519 [4]	AY682482 [4]
		Limnochromis abeelei	LT	AY682533 [4]	AY682496 [4]
		Limnochromis auritus	LT	AY682536 [4]	AY682499 [4]
		Limnochromis staneri	LT	AY682538 [4]	AY682501 [4]
		Reganochromis calliurus	LT	AY682544 [4]	AY682506 [4]
		Tangachromis dhansi	LT	n.a.	n.a.
		Triglachromis otostigma	LT	AY682546 [4]	AY682508 [4]
**Orthochromini**		*Orthochromis malagaraziensis*	Malagarazi river, TZ	AF398232 [9]	AF400714 [9]
		Orthochromis rugufuensis	Rugufu river, TZ	AY930050 [10]	AY929945 [10]
		Orthochromis uvinzae	Malagarazi river, TZ	AY930048 [10]	AY929943 [10]
**Perissodini**	4/9	*Haplotaxodon microlepis*	LT	AY682529 [4]	AY682492 [4]
		Haplotaxodon trifasciatus	LT	AY682531 [4]	AY682494 [4]
		Perissodus microlepis	LT	AF317265 [5]	AF400730 [9]
		Plecodus eccentricus	LT	n.a.	n.a.
		Plecodus elaviae	LT	n.a.	n.a.
		Plecodus paradoxus	LT	n.a.	n.a.
		Plecodus multidentatus	LT	n.a.	n.a.
		Plecodus straeleni	LT	AF398221 [9]	AF400731 [9]
		Xenochromis hecqui	LT	n.a.	n.a.
**Tilapiini**	1/?	*Oreochromis tanganicae*	LT	AF317240 [9]	AY929940 [10]
**Trematocarini**	2/9	*Trematocera caprti*	LT	n.a.	n.a.
		Trematocera kufferathi	LT	n.a.	n.a.
		Trematocera macrostoma	LT	AY663715 [7]	AY663760 [7]
		Trematocera marginatum	LT	n.a.	n.a.
		Trematocera nigrifrons	LT	n.a.	n.a.
		Trematocara unimaculatum	LT	AF317268 [5]	AY663759 [7]
		Trematocera stigmaticum	LT	n.a.	n.a.
		Trematocera variable	LT	n.a.	n.a.
**Tropheini**	16/26	*Ctenochromis horei*	LT	AY930100 [10]	AY301952 [12]
		Gnathochromis pfefferi	LT	AY682518 [4]	AY682481 [4]
		Limnotilapia dardennii	LT	DQ093109 [2]	AY 301957 [12]
		Lobochilotes labiatus	LT	U07254 [8]	U01110 [8]
		Petrochromis ephippium	LT	n.a.	n.a.
		Petrochromis famula	LT	n.a.	n.a.
		Petrochromis fasciolatus	LT	n.a.	n.a.
		Petrochromis macrognathus	LT	AY930068 [10]	AY929963 [10]
		Petrochromis orthognathus	LT	U07262 [8]	U01111 [8]
		Petrochromis polydon	LT	n.a.	n.a.
		Petrochromis trewavasae	LT	n.a.	n.a.
		Simochromis curvifrons	LT	n.a.	n.a.
		Simochromis babaulti	LT	DQ093110 [2]	AF400736 [9]
		Simochromis diagramma	LT	AY930087 [10]	AY929974 [10]
		Simochromis loocki	LT	n.a.	n.a.
		Simochromis marginatus	LT	AY930088 [10]	AY929975 [10]
		Simochromis margaretae	LT	n.a.	n.a.
		Simochromis pleurospilus	LT	n.a.	n.a.
		Tropheus annectens	LT	n.a.	n.a.
		Tropheus brichardi	LT	AY930086 [10]	AY930022 [10]
		Tropheus duboisi	LT	AY930085 [10]	AY930021 [10]
		Tropheus moorii I	LT	AY930066 [10]	AY929961 [10]
		Tropheus moorii II	LT	AY930067 [10]	AY929962 [10]
		Tropheus moorii III	LT	AY930091 [10]	AY929978 [10]
		Tropheus moorii IV	LT	AY930093 [10]	AY929980 [10]
		Tropheus polli	LT	AY930084 [10]	AY929971 [10]
