# Supplementary material for: Tempo and Mode of Diversification of Lake Tanganyika Cichlid Fishes
Source: PLoS One. 2008 Mar 5;3(3):e1730. doi: 10.1371/journal.pone.0001730 (PMC2248707; doi:10.1371/journal.pone.0001730)
Supplement: Table S2 — Speciation rate statistics for the LT cichlid radiation, major lineages and constituent tribes. (0.08 MB PDF) [file pone.0001730.s003.pdf]

Table S2. Speciation rate statistics for the LT cichlid radiation, major lineages and constituent tribes. The net diversification rate was estimated using the Kendal-Moran estimator Nee's lambda (λ) [16], based on birth death model [17] implemented in APE using the Yule prior [18]. Net diversification rate statistics are also obtained using the proportional-to-distinguishable prior for comparison [18]. As these statistics are similar to those obtained through the Yule prior a single table is included here for reference purposes. Confidence intervals reflect the uncertainty from the birth-death model and variation in date estimates across the 1000 Bayesian samples. Log likelihood ratio tests comparing birth and birth-death models confirmed no significant evidence for non-zero extinction rates throughout. γ =gamma statistic [19]. Rates are estimated for all species sampled in this study assuming 12 Myr and 28 Myr calibrations [20], and incorporating missing species (see Figure S1 for placements). Rate statistics are calculated for endemic cichlids only, with all non-endemics dropped from analyses.

a) Yule prior with missing species added (12 Myr calibration)

	Diversification Rate			Extinction ratio (b/d)			LLRT χ^2	Pybus & Harvey test	
Clade	Mean	CI.lower	CI.upper	Mean	CI.lower	CI.upper		γ	p
LT flock	0.355	0.308	0.407	0	0	0.135	1	-2.253	0.058
P-lineage	0.394	0.339	0.455	0	0	0.119	1	-3.199	0.007
C-lineage	0.395	0.317	0.484	0	0	0.219	1	-1.710	0.146
L-lineage	0.388	0.313	0.475	0	0	0.178	1	-2.768	0.018
Bathybatini	0.187	0.067	0.404	0.04	0.002	0.678	1	-0.886	0.425
Cyprichromini	0.356	0.205	1.375	NA	NA	NA	NA	-0.123	0.692
Ectodini	0.355	0.241	0.500	0	0	0.334	1	-1.948	0.075
Lamprologini	0.406	0.325	0.500	0	0	0.174	1	-3.100	0.008
Limnochromini	0.359	0.180	0.630	0.012	0.002	0.543	0.99	-1.278	0.265
Tropheini	0.599	0.390	0.872	0	0	0.365	1	-2.003	0.101
LM & LV min	NA	NA	NA	NA	NA	NA	NA	NA	NA
LM & LV max	NA	NA	NA	NA	NA	NA	NA	NA	NA

b) Yule prior (12 Myr calibration)

	Diversification Rate			Extinction ratio (b/d)			LLRT χ^2	Pybus & Harvey test	
Clade	Mean	CI.lower	CI.upper	Mean	CI.lower	CI.upper		γ	p
LT flock	0.312	0.265	0.365	0	0	0.136	1	-2.900	0.009
P-lineage	0.350	0.295	0.411	0	0	0.120	1	-3.873	0.000
C-lineage	0.335	0.261	0.423	0	0	0.207	1	-2.693	0.014
L-lineage	0.358	0.281	0.449	0	0	0.199	1	-2.735	0.013
Bathybatini	0.189	0.068	0.409	0.035	0.002	0.676	1	-0.902	0.415
Cyprichromini	0.357	0.205	1.378	NA	NA	NA	NA	-0.129	0.693
Ectodini	0.317	0.209	0.458	0	0	0.315	1	-2.732	0.011
Lamprologini	0.380	0.296	0.478	0	0	0.192	1	-3.080	0.005
Limnochromini	0.307	0.132	0.593	0.001	0	0.595	1	-1.264	0.248
Tropheini	0.461	0.260	0.747	0	0	0.428	1	-2.356	0.029
LM & LV min	2.035	1.645	3.135	NA	NA	NA	NA	NA	NA
LM & LV max	2.105	1.715	3.205	NA	NA	NA	NA	NA	NA

c) Yule prior with missing species added (28 Myr calibration)

	Diversification Rate			Extinction ratio (b/d)			LLRT χ^2	Pybus & Harvey test	
Clade	Mean	CI.lower	CI.upper	Mean	CI.lower	CI.upper		γ	p
LT flock	0.145	0.126	0.166	0	0	0.128	1	-2.488	0.027
P-lineage	0.160	0.137	0.184	0	0	0.117	1	-3.303	0.003
C-lineage	0.160	0.129	0.196	0	0	0.217	1	-1.741	0.126
L-lineage	0.160	0.127	0.192	0	0	0.175	1	-2.872	0.011
Bathybatini	0.077	0.027	0.165	0.013	0	0.662	1	-1.036	0.340
Cyprichromini	0.153	0.088	0.588	NA	NA	NA	NA	-0.406	0.637
Ectodini	0.146	0.099	0.205	0	0	0.332	1	-1.987	0.064
Lamprologini	0.164	0.131	0.201	0	0	0.173	1	-3.140	0.005
Limnochromini	0.141	0.071	0.248	0.007	0	0.541	1	-1.270	0.277
Tropheini	0.237	0.154	0.344	0	0	0.374	1	-1.809	0.142
LM & LV min	NA	NA	NA	NA	NA	NA	NA	NA	NA
LM & LV max	NA	NA	NA	NA	NA	NA	NA	NA	NA

d) Yule prior (28 Myr calibration)

	Diversification Rate			Extinction ratio (b/d)			LLRT χ^2	Pybus & Harvey test	
Clade	Mean	CI.lower	CI.upper	Mean	CI.lower	CI.upper		γ	P
LT flock	0.127	0.107	0.148	0	0	0.128	1	-3.207	0.002
P-lineage	0.141	0.119	0.166	0	0	0.118	1	-4.013	0.000
C-lineage	0.135	0.105	0.170	0	0	0.205	1	-2.738	0.011
L-lineage	0.144	0.113	0.181	0	0	0.195	1	-2.866	0.007
Bathybatini	0.077	0.028	0.165	0	0	0.660	1	-1.047	0.333
Cyprichromini	0.153	0.088	0.590	NA	NA	NA	NA	-0.403	0.634
Ectodini	0.130	0.085	0.187	0	0	0.313	1	-2.795	0.008
Lamprologini	0.152	0.119	0.192	0	0	0.190	1	-3.151	0.003
Limnochromini	0.120	0.051	0.231	0	0	0.595	1	-1.238	0.252
Tropheini	0.181	0.102	0.293	0	0	0.436	1	-2.136	0.047
LM & LV min	0.814	0.658	1.255	NA	NA	NA	NA	NA	NA
LM & LV max	0.842	0.686	1.283	NA	NA	NA	NA	NA	NA

e) Proportional-to-distinguishable prior with missing species added (12 Myr calibration)

	Diversification Rate			Extinction ratio (b/d)			LLRT χ^2	Pybus & Harvey test	
Clade	Mean	CI.lower	CI.upper	Mean	CI.lower	CI.upper		γ	p
LT flock	0.256	0.222	0.293	0	0	0.096	1	-4.249	0.000
P-lineage	0.271	0.233	0.313	0	0	0.097	1	-4.537	0.000
C-lineage	0.273	0.220	0.335	0	0	0.182	1	-2.755	0.016
L-lineage	0.265	0.214	0.324	0	0	0.159	1	-3.495	0.002
Bathybatini	0.150	0.054	0.322	0	0	0.646	1	-1.259	0.241
Cyprichromini	0.247	0.142	0.952	NA	NA	NA	NA	-0.532	0.577
Ectodini	0.256	0.174	0.361	0	0	0.319	1	-2.280	0.041
Lamprologini	0.271	0.217	0.333	0	0	0.163	1	-3.548	0.002
Limnochromini	0.223	0.112	0.391	0.005	0	0.534	0.99	-1.397	0.226
Tropheini	0.372	0.242	0.541	0.001	0	0.373	1	-1.862	0.133
LM & LV min	NA	NA	NA	NA	NA	NA	NA	NA	NA
LM & LV max	NA	NA	NA	NA	NA	NA	NA	NA	NA
