# Supplementary material for: Tempo and Mode of Diversification of Lake Tanganyika Cichlid Fishes
Source: PLoS One. 2008 Mar 5;3(3):e1730. doi: 10.1371/journal.pone.0001730 (PMC2248707; doi:10.1371/journal.pone.0001730)
Supplement: Text S1 — Lake Tanganyika cichlid phylogeny Key-innovation test Supporting references (0.07 MB PDF) [file pone.0001730.s004.pdf]

Supporting Text

Lake Tanganyika cichlid phylogeny

The inclusion of considerably more species than previous studies [2], [9], recovers trees that reveal a high level of taxonomic congruence under both ML and BI methods (-ln L 41348.47, -ln L 41352.22 respectively), so that neither tree can be rejected as a significantly worse fit to the data, based on the AU tests (p = 0.563). Quantitative support for relationships is good, with BPP performing better than BS (Figure S5). Our results recover a single clade (P = principal radiation) containing the majority of taxa (~ 92%), with tribes Bathybatini, Trematocarini, Hemibatini, Boulengerochromini and Tilapiini recovered as successive sister groups. Within the principal radiation (P, Figure S5) two main clades are recovered: the C-lineage [2] ('H-lineage') [9,], [17] and a clade composed of Lamprologini and Eretmodini, defined here as the L-lineage, also supporting the ncDNA placement of these tribes [2]. The monophyly of all tribes are recovered with maximum branch support, supporting previous morphological and molecular work. Our tree also recovers two clades within the C-Lineage, however, while these are well-supported by BPP (ignoring the placement of *Cyphotilapia frontosa*), the reverse is true of BS. With the exception of the sister relationship of Orthochromini and Haplochromini (the latter tribe includes LM and LV radiations), there appears to be no common consensus of the relationships within the C-lineage when considering our data and others e.g. [2], [9].

Key-innovation test

As higher diversification rates may be the result of a newly evolved morphological or ecological character, namely a key innovation [18], we tested the trait shell-brooding, unique to lamprologines, using the likelihood key-innovation test [19] to determine the significance of this character. Both obligate and facultative species are included (table S2). We found a marginally significant result (p = 0.045), when comparing lamprologines to their sister group, the Eretmodini, which comprise the L-lineage, for only those species sampled in this study. The results suggest that this character may have promoted lineage diversification within the Lamprologini.

Supporting references

Table S1

1. **Brandstätter A, Salzburger W, Sturmbauer C** (2005) Mitochondrial phylogeny of the Cyprichromini, a lineage of open-water cichlid fishes endemic to Lake Tanganyika, East Africa. Mol Phylogenet Evol 34: 382-391.
2. **Clabaut C, Salzburger W, Meyer A** (2005). Comparative phylogenetic analyses of the adaptive radiation of Lake Tanganyika cichlid fish: Nuclear sequences are less homoplasious but also less informative than mitochondrial DNA. J Mol Evol 61: 666-681.
3. **Day JJ, Santini S, Garcia-Moreno J** (2007) Phylogenetic relationships of the lake Tanganyika cichlid tribe lamprologini: The story from mitochondrial DNA. Mol Phylogenet Evol 45: 629-642.
4. **Duftner N, Koblmüller S, Sturmbauer C** (2005) Evolutionary relationships of the Limnochromini, a tribe of benthic deepwater Cichlid fish endemic to Lake Tanganyika, East Africa. J Mol Evol 60: 277–289.
5. **Klett V, Meyer A** (2002) What, if anything, is a Tilapia? - Mitochondrial ND2 phylogeny of tilapiines and the evolution of parental care systems in the African cichlid fishes. Mol Biol Evol 19: 865–883
6. **Koblmüller S, Salzburger W, Sturmbauer C** (2004) Evolutionary relationships in the sand-dwelfing cichlid lineage of lake Tanganyika suggest multiple colonization of rocky habitats and convergent origin of biparental mouthbrooding. J Mol Evol 58: 79–96.
7. **Koblmüller S, Duftner N, Katongo C, Phiri H, Sturmbauer C** (2005) Ancient divergence in bathypelagic Lake Tanganyika deepwater cichlids: Mitochondrial phylogeny of the tribe Bathybatini. J Mol Evol 60: 297-314.
8. **Kocher TD, Conroy JA, McKaye KR, Stauffer JR, Lockwood SF** (1995) Evolution of NADH dehydrogenase subunit 2 in east African cichlid fish. Mol Phylogenet Evol 4: 420-432.
9. **Salzburger W, Meyer A, Baric S, Verheyen E, Sturmbauer C** (2002) Phylogeny of the Lake Tanganyika Cichlid species flock

and its relationship to the Central and East African Haplochromine Cichlid fish. Syst Biol 51: 1–23.

10. **Salzburger W, Mack T, Verheyen E, Meyer A** (2005) Out of Tanganyika: Genesis, explosive speciation, key-innovations and phylogeography of the haplochromine cichlid fishes. BMC Evol Biol 5: 17.
11. **Schelly R, Salzburger W, Koblmüller S, Duftner N, Sturmbauer C** (2006) Phylogenetic relationships of the lamprologine cichlid genus Lepidiolamprologus (Teleostei : Perciformes) based on mitochondrial and nuclear sequences, suggesting introgressive hybridization. Mol Phylogenet Evol 38:426-438.
12. **Sturmbauer C, Hainz U, Baric S, Verheyen E, Salzburger S** (2003) Evolution of the tribe Tropheini from Lake Tanganyika: synchronized explosive speciation producing multiple evolutionary parallelism. Hydrobiol 500: 51-64.
13. **Sturmbauer C, Meyer A** (1993) Mitochondrial phylogeny of the endemic mouthbrooding lineages of cichlid fishes from lake tanganyika in eastern africa. Mol Biol Evol 10: 751–768.
14. **Rüber L, Verheyen E, Meyer A** (1999) Replicated evolution of trophic specializations in an endemic cichlid fish lineage from Lake Tanganyika. Proc Natl Acad Sci USA 96: 10230-10235.
15. **Genner MJ, Seehausen O, Cleary DFR, Knight ME, Michel E, Turner GF** (2004) How does the taxonomic status of allopatric populations influence species richness within African cichlid fish assemblages? J Biogeogr 31: 93-102.

Table S2

16. **Nee S** (2001) Inferring speciation rates from phylogenies. Evolution 55: 661–668.
17. **Nee S, May RM, Harvey PH** (1994) The reconstructed evolutionary process. Phil Trans R Soc Lond B 344: 305-311.
18. **Paradis E, Claude J, Strimmer K** (2004) APE: Analyses of Phylogenetics and Evolution in R language. Bioinform 20: 289-290.
19. **Pybus OG, Harvey PH** (2000) Testing macro-evolutionary models using incomplete molecular phylogenies. Proc. R. Soc. Lond. B 267: 2267–2272.
20. **Genner MJ, Seehausen O, Lunt DH, Joyce, DA, Shaw PW, Carvalho, GR, Turner GF** (2007) Age of cichlids: New dates for ancient lake fish radiations. Mol Biol Evol 24: 1269-1282.

Supporting text

17 **Nishida M** (1991) Lake Tanganyika as an evolutionary reservoir of old lineages of East-African cichlid fishes - inferences from allozyme data. Experientia 47: 974-979.

18. **Maynard Smith J, Szathmáry E** (1995) The Major Transitions in Evolution Oxford: Oxford University Press.
19. **Ree RH** (2005) Detecting the historical signature of key innovations using stochastic models of character evolution and cladogenesis. Evolution 59: 257-265.
